# Copy number abnormalities in new or progressive ‘neurocutaneous melanosis’ confirm it to be primary CNS melanoma

**DOI:** 10.1007/s00401-016-1651-0

**Published:** 2016-12-08

**Authors:** Veronica A. Kinsler, Satyamanaasa Polubothu, J. Eduardo Calonje, W. Kling Chong, Dominic Thompson, Thomas S. Jacques, Deborah Morrogh

**Affiliations:** 1 Paediatric Dermatology, Great Ormond Street Hospital for Children NHS Foundation Trust, London, UK; 2 Genetics and Genomic Medicine, UCL Institute of Child Health, London, WC1N 1EJ UK; 3 Dermatopathology, St John’s Institute of Dermatology, Guys and St Thomas’ NHS Foundation Trust, London, UK; 4 Paediatric Neuroradiology, Great Ormond Street Hospital for Children NHS Foundation Trust, London, UK; 5 Paediatric Neurosurgery, Great Ormond Street Hospital for Children NHS Foundation Trust, London, UK; 6 Developmental Biology and Cancer Programme, UCL Institute of Child Health, London, UK; 7 Department of Histopathology, Great Ormond Street Hospital for Children NHS Foundation Trust, London, UK; 8 Regional Genetics Laboratory, Great Ormond Street Hospital for Children, London, UK

The term ‘neurocutaneous melanosis’ was coined in 1861 as a post-mortem description of multiple congenital melanocytic naevi (CMN) and fatal diffuse leptomeningeal melanocytosis [5]. The congenital phenotype of this disease is caused by post-zygotic mutations in the gene *NRAS* in 80% of cases [3], and progression to cutaneous melanoma involves further mutations [1, 3, 6]. We have previously argued that there should be a distinction made between congenital abnormalities of the CNS and acquired primary CNS melanoma [7], a situation which can be considered analogous to the presence of benign congenital naevi on the skin, and the risk of development of cutaneous melanoma. The commonest congenital CNS lesion is intraparenchymal melanosis, which has highly characteristic MRI features, and even if symptomatic does not carry a poor prognosis in terms of life expectancy and does not require biopsy [7]. However, rarer lesions cause more diagnostic uncertainty, and in particular, the term ‘leptomeningeal melanocytosis’ currently includes both stable congenital disease, and either newly acquired or clinically and radiologically progressive fatal disease [4, 7]. The problem with distinguishing benign from malignant leptomeningeal disease other than by clinical outcome has been that histological studies do not always demonstrate malignant morphology or leptomeningeal invasion of the underlying parenchyma, despite being ultimately fatal. Histology, however, continues to form a key part of any assessment, including for distinguishing melanoma from the very rare occurrence of other CNS tumours in this condition.

Seminal work on benign and malignant tumours arising within cutaneous CMN established that in general there is a difference in chromosomal copy number patterns between CMN, benign proliferative nodules, and cutaneous melanoma arising in CMN, a study which included one metastatic CNS sample [2]. To date, however, there has been no published data on chromosomal copy number pattern in primary CNS melanoma arising in children with CMN, and we sought to establish the value of this test.

DNA was extracted directly from six fresh CNS biopsy specimens using standard methods. These samples were taken prospectively from all cases of suspected primary CNS melanoma seen in our tertiary referral service over a period of six years. Five were from patients with a clinico-radiological diagnosis of newly acquired or progressive melanotic CNS disease, and one from a rare case of diffuse leptomeningeal melanocytosis which has been clinically only very slowly progressive, and radiologically stable over 11 years after initial leptomeningeal spread during the first year of life. All six were *NRAS* codon 61 mutation positive, and *BRAF* codon 600 wild-type. In one of the six patients there was classical progressive diffuse melanotic leptomeningeal disease, without skin disease (patient 1, supplementary Table 1). This patient was included as the leptomeningeal disease was indistinguishable clinically and genetically from that seen in patients with CMN. Whole genome array comparative genomic hybridization (CGH) was performed using Roche Nimblegen 135K oligonucleotide arrays or Affymetrix CytoScan 750K and commercial sex-matched pooled controls.

All five biopsies from patients with a clinico-radiological diagnosis of CNS melanoma based on tumour behavior (patients 1–5) showed large gains and/or losses of parts of chromosomes, with a similar pattern to known findings in cutaneous melanoma in CMN patients [2] (Fig. 1, supplementary Tables 1 and 2), and tragically, all five children have succumbed to their disease. In the patient with stable diffuse leptomeningeal melanocytosis, however, we found a normal chromosomal copy number pattern and the patient continues to be clinically stable.

**Fig. 1:**
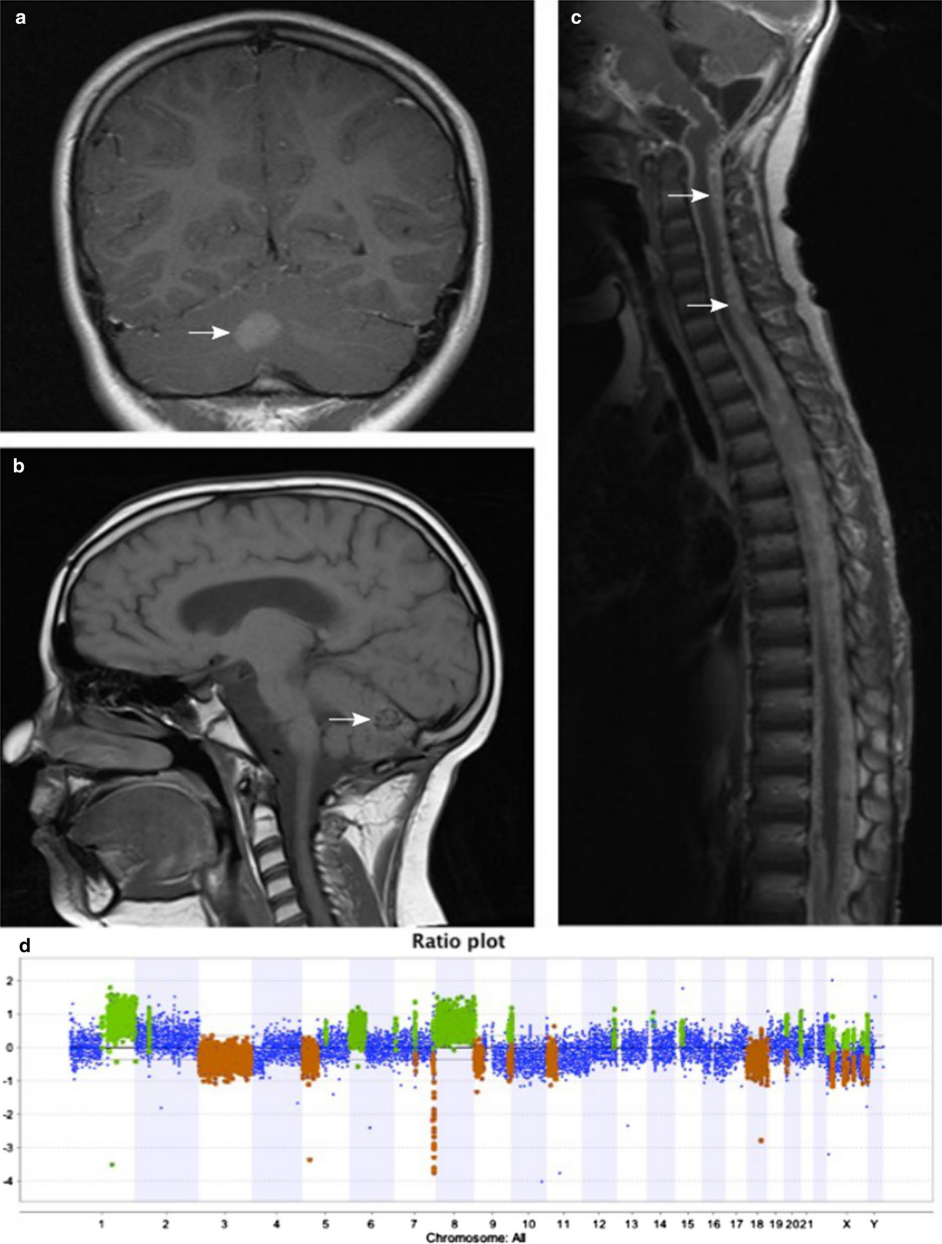
MRI of patient 2 demonstrating melanoma within the cerebellum (**a**, **b**) and physically unconnected diffuse leptomeningeal melanoma (**c**). Cerebellar melanoma showing multiple large gains and losses on array CGH (**d**)

Whole genome copy number abnormalities in newly acquired, or clinico-radiologically progressive melanotic disease in the CNS seem to mirror those described in cutaneous melanoma, with a characteristic signature of multiple gains and losses of parts of chromosomes seen in fatal cases even where histology was not clearly malignant. These can, therefore be considered as primary CNS melanoma. On the other hand, no copy number abnormalities were seen in the one case of stable leptomeningeal disease. This supports the use of copy number measurement as an adjunct to early screening MRI of the CNS to characterize the congenital neurological phenotype, and of *NRAS* and *BRAF* genotyping and histopathology where biopsies of suspected primary CNS melanoma are taken.

## Electronic supplementary material

Below is the link to the electronic supplementary material.

Supplementary material 1 (DOCX 61 kb)

Supplementary material 2 (XLSX 50 kb)
