# Supplementary material for: Copy number abnormalities in new or progressive ‘neurocutaneous melanosis’ confirm it to be primary CNS melanoma
Source: Acta Neuropathol. 2016 Dec 8;133(2):329–31. doi: 10.1007/s00401-016-1651-0 (PMC5250663; doi:10.1007/s00401-016-1651-0)
Supplement: Supplementary file 1 — Supplementary material 1 (DOCX 61 kb) [file 401_2016_1651_MOESM1_ESM.docx]

**Supplementary Table 1**

Clinical and genetic details of patients with suspected primary CNS melanoma seen in our department, including one patient with no cutaneous lesions, and one patient with clinically-stable diffuse leptomeningeal disease. All tissue samples were wild type for the codon 600 hotspot in *BRAF*.

PAS – projected adult size

*full details of copy number results available in supplementary table 2

| **Patient**  **number** | **Sex** | **Age at diagnosis (years)** | **Outcome** | **Screening MRI CNS under 6 months** | **Primary melanoma site** | **CMN classification** | **Tissue *NRAS* hotspot genotype (codons 12,13,61)** | **Tissue whole genome large (>1MB) copy number changes** | **Histology of CNS biopsy** |
| --- | --- | --- | --- | --- | --- | --- | --- | --- | --- |
| 1 | Female | 1.5y | Death aged 2y | Not done | CNS, diffuse leptomeningeal melanoma | No CMN | c.181C>A; p.Q61K | Gain 1q2.13;  LOH 18p11.32 | Leptomeningeal melanoma |
| 2 | Female | 9.7y | Death, age 10.2y | Normal | CNS, solid tumour in cerebellum, plus diffuse leptomeningeal melanoma | Multiple CMN, largest >60cm PAS, bathing trunk, 100-200 naevi in total | c.181C>A; p.Q61K | *Gain 1q, 6p, 8, 12q, 15q;  LOH 3, 5p, 11p, 18 | Cerebellar biopsy - Primary melanocytic tumour favouring diagnosis of melanoma |
| 3 | Male | 1.5y | Death age 2.3y | Complex congenital neurological disease | CNS, diffuse leptomeningeal melanoma | Multiple CMN, largest in bathing trunk distribution, PAS >60cm, 100-200 naevi in total | c.181C>A; p.Q61K | Gain 6p25.3-p12.1 | Leptomeningeal melanocytosis |
| 4 | Male | 4.0y | Death, age 4.6y | Complex congenital neurological disease | CNS, diffuse leptomeningeal melanoma | Multiple CMN, largest neck and upper back, cape, PAS 20-40cm, 100-200 naevi in total | c.181C>A; p.Q61K | *LOH 16p, 16q, Xp, Xq | Leptomeningeal melanoma |
| 5 | Female | 1.8y | Death age 2.2y | Complex congenital neurological disease | CNS, diffuse leptomeningeal melanoma | Multiple CMN, no clearly larger naevus. >400 naevi in total. | c.181C>A; p.Q61K | *Gains 1p, 1q, 6p;  LOH 2q, Xp | Leptomeningeal melanoma |
| 6 | Female | N/A | Alive aged 12y, moderate neurodevelopmental delay, seizures, stable compressive spinal cord signs | Complex congenital neurological disease | No melanoma | Multiple CMN, largest neck and upper back, cape, PAS 40-60cm, 100-200 naevi in total | c.181C>A; p.Q61K | No large gains or losses | Leptomeningeal melanocytosis |

**Supplementary table 2**

Details of large copy number abnormalities (>1MB) by patient number from supplementary table 1
